# CWC22-dependent pre-mRNA splicing and eIF4A3 binding enables global deposition of exon junction complexes

**DOI:** 10.1093/nar/gkv320

**Published:** 2015-04-13

**Authors:** Anna-Lena Steckelberg, Janine Altmueller, Christoph Dieterich, Niels H. Gehring

**Affiliations:** 1 Institute for Genetics, University of Cologne, D-50674 Cologne, Germany; 2 Cologne Center for Genomics (CCG), University of Cologne, D-50931 Cologne, Germany; 3 Institute of Human Genetics, University of Cologne, D-50931 Cologne, Germany; 4 Max Planck Institute for Biology of Ageing, Joseph-Stelzmann-Straße 9b, D-50931 Cologne, Germany

## Abstract

In metazoan cells, spliced mRNAs are marked by the exon junction complex (EJC), a multi-protein complex that serves as a key regulator of post-transcriptional mRNA metabolism. Deposition of EJCs on mRNA is intimately linked to the splicing process. The spliceosomal protein CWC22 directly binds the core EJC-protein eIF4A3, guides it to the spliceosome and initiates EJC assembly. In addition, CWC22 is involved in the splicing process itself, but the molecular details of its dual function remain elusive. Here we analyze the mechanisms, by which CWC22 co-regulates pre-mRNA splicing and EJC assembly. We show that the core of CWC22 is sufficient to mediate both pre-mRNA splicing and EJC assembly. Nonetheless, both processes can be functionally uncoupled with an eIF4A3-binding deficient mutant of CWC22, which impedes EJC assembly. A C-terminal domain of CWC22 strongly enhances its spliceosomal interaction and likely regulates its function. High-throughput RNA-sequencing identifies global defects of pre-mRNA splicing and downregulation of diverse gene expression pathways in CWC22-depleted cells. We propose a model, in which CWC22 represents an integral component of the spliceosome and orchestrates pre-mRNA splicing and eIF4A3 binding to achieve global assembly of exon junction complexes.

## INTRODUCTION

Pre-mRNA processing in higher eukaryotes is closely linked to the remodeling of the messenger ribonucleoprotein particle (mRNP). An important component of the post-splicing mRNP is the exon junction complex (EJC), a multi-protein complex that is deposited on nascent mRNAs during splicing (1). The core of the EJC consists of the four proteins eIF4A3 (DDX48), MAGOH, Y14 (RBM8A) and Barentsz (BTZ, also called CASC3 or MLN51), which stably bind spliced mRNA in close proximity to exon–exon junctions (2–4). During different steps of gene expression, this tetrameric EJC core recruits accessory proteins that influence the fate of the mRNA (5–7). By marking (prior) splice sites, EJCs are instrumental to identify transcripts with premature translation termination codons (PTCs), which are degraded by a quality control mechanism called nonsense mediated mRNA decay (NMD) (8,9). Moreover, EJCs stimulate the translation of spliced mRNAs (10,11), and regulate the cytoplasmic localization of *oskar* mRNA in *Drosophila* oocytes (12). Recently it was shown that EJCs also directly influence the splicing of a subset of transcripts in *Drosophila* and human cells (13–17).

The RNA-interaction of the EJC is mediated by eIF4A3, an ATPase of the DEAD-box family (18). The MAGOH-Y14 heterodimer binds to eIF4A3, inhibits both its ATPase activity and dissociation from RNA and thereby stabilizes the EJC on the mRNA (19). Since eIF4A3 mainly contacts the ribose-phosphate backbone of the mRNA, its binding is independent of the nucleotide sequence. Despite this lack of sequence-specificity, EJCs almost invariantly assemble 20–24 nucleotides (nt) upstream of exon–exon junctions (1). This very precise EJC deposition suggests that the spliceosome itself regulates the assembly of EJCs on the mRNA.

The spliceosomal protein CWC22 was recently identified as a direct interaction partner of eIF4A3 (20–22). The interaction is mediated by the MIF4G domain of CWC22 and structurally resembles other complexes of MIF4G domains and DEAD-box proteins, e.g. eIF4G and eIF4A (23). Binding to CWC22 recruits eIF4A3 to the spliceosome and initiates EJC assembly at an early step of splicing. Notably, CWC22 is not required for the assembly of EJCs from recombinant components *in vitro*, indicating that the function of CWC22 during EJC formation is directly linked to the splicing process (20). CWC22 is also involved in pre-mRNA splicing itself and unspliced pre-mRNAs accumulate in CWC22-depleted cells (20,22). The splicing defect can be rescued by a CWC22 construct comprising of MIF4G and adjacent MA3 domain, demonstrating that the MIF4G domain is involved in both pre-mRNA splicing and EJC deposition (20). However, the molecular basis for this dual function of CWC22 remains elusive.

Here, we investigate the interrelation between CWC22, EJC and the spliceosome with a combination of *in vitro* splicing and *in vivo* complementation assays. We observe a sequential association of CWC22 and eIF4A3 with the splicing machinery and identify domains that regulate the spliceosomal interaction of CWC22. Moreover, we demonstrate that neither the recruitment of CWC22 to the spliceosome, nor its function during pre-mRNA splicing, requires the interaction with eIF4A3. The depletion of CWC22 causes global defects of pre-mRNA splicing and establishes a role of CWC22 as a general splicing factor. Together, our data provide the basis for a refined model of EJC assembly.

## MATERIALS AND METHODS

### Plasmids

Constructs for protein expression in mammalian cell culture were inserted into the pCI-neo vector (Promega) with an N-terminal FLAG (DYKDDDDK) tag. Templates for *in vitro* transcription of radiolabeled mRNA were EcoRI/BamHI cloned into pGEM-4Z (Promega). MINX, MINX Δi and the expression vectors for FLAG-Y14, FLAG-eIF4A3, FLAG-CWC22 and their respective mutants were described previously (20,24). CWC22 deletion mutants 110–908, 340–908, 1–769 and Δ669–759 were polymerase chain reaction (PCR) amplified and inserted into pCI-neo-FLAG. AdML-PT60 and AdML-PT60/e1(18) were generated by PCR amplification of a synthetic DNA. All constructs were verified by DNA sequencing.

### Cell culture, plasmid transfections and siRNA transfections

HEK293 cells and HEK293 Flp-In^TM^ T-Rex^TM^ cells (Life Technologies) were grown in Dulbecco's Modified Eagle Medium (DMEM), supplemented with 1% penicillin/streptomycin and 10% FBS at 37°C and 5% CO_2_. HEK293 Flp-In^TM^ T-Rex^TM^ cells stably expressing tetracycline-inducible FLAG-CWC22 constructs were generated according to the manufacturer's instructions and protein expression was induced by culturing the cells in the presence of 1 μg/ml doxycycline for 48 h. Transfection with plasmid DNA was performed as previously described (24). For complementation assays, 1.8x 10^5^ HEK 293 Flp-IN^TM^ T-Rex^TM^ cells were seeded in 6-well plates containing 1 μg/m doxycycline 24 h prior to transfection. SiRNAs (10 nM) were transiently transfected using Lipofectamine RNAiMax reagent (Life Technologies) and cells were harvested 48 h post-transfection. The siRNA target sequences were CWC22 (5′-AAAGTAGTGTGGCACAGATAA-3′), Y14 (5′-ATATGAAACATACAAGGAA-3′) and luciferase (5′-AACGUACGCGGAAUACUUCGATT-3′). siRNA resistant CWC22 constructs were generated by replacing the siRNA targeting sequence (codons 2–9 of the CWC22 ORF) by the resistant sequence 5′-AAA TCA TCA GTG GCC CAA ATC AAA-3′.

### RNA extraction and analysis

Total RNA was extracted with Isol-RNA lysis reagent (5 Prime) according to the manufacturer's instructions. When proteins were analyzed in parallel to RNA, proteins were extracted from the phenol phase using acetone precipitation according to the manufacturer's protocol.

Quantitative RT-PCR measuring SYBR Green incorporation was used to quantify pre-mRNA and mRNA expression levels of GAPDH and mRNA expression levels of SC35 and GAS5. The expression levels of SC35 and GAS5 were normalized to the housekeeping gene TATA box binding protein (TBP). Primer sequences were

5′-GAGTCAACGGATTTGGTCGT-3′ and 5′-TTGATTTTGGAGGGATCTCG-3′ (GAPDH mRNA)

5′-GAGCTGGGGAATGGGACT-3′ and 5′-TGATGGCATGGACTGTGG-3′ (GAPDH pre-mRNA)

5′-TGCACAGGAGCCAAGAGTGAA-3′ and 5′-CACATCACAGCTCCCCACCA-3′ (hTBP)

5′-GGCGTGTATTGGAGCAGATGTA-3′ and 5′-CTGCTACACAACTGCGCCTTTT-3′ (SC35)

5′-GGTATGGAGAGTCGGCTTGA-3′ and 5′-GCACTCTAGCTTGGGTGAGG-3′ (GAS5)

5′-GGGATGGAAAGTCACCCGTA-3′ and 5′-AGAACACCAGTCTCCACTCG-3′ (EEF1A1 mRNA)

5′-GGGAATGGCGATTTCATGCT-3′ and 5′-GGACGAGTTGGTGGTAGGAT-3′ (EEF1A1 intron 4)

5′-AAGTTGGCTGTAAACAAAGTTGA-3′ and 5′-AAGTTGGCTGTAAACAAAGTTGA-3′ (EEF1A1 intron 5)

5′-TGAGACTTTGGATTTGCACTGA-3′ and 5′-AACAATGGCAGCATCACCAG-3′ (EEF1A1 intron 6)

5′-GTCAAGGATGTTCGTCGTGG-3′ and 5′-ACCCAAAGTACTGTTCAGTTGT-3′ (EEF1A1 intron 7)

5′-TGGTTGGTGATGGTGGTACT-3′ and 5′-CCATCTCTCAGTCCACCGAA-3′ (RAN mRNA)

5′-CCATCTCTCAGTCCACCGAA-3′ and 5′-ACGATGCGATTGAGTGGATG-3′ (RAN intron 3)

5′-ATTGTGGCCACTTTGCTGTT-3′ and 5′-CCCACTCATCTCCTTCAGCA-3′ (RAN intron 6)

5′-CCCCTACATCCCTTCAC-3′ and 5′-GGGTGATGTGGGACTAT-3′ (POLR2A)

### Immunoblot analysis and immunoprecipitation

Cells were harvested in lysis buffer (50 mM Tris (pH 7.2), 150 mM NaCl, 0.5% Triton X-100, + protease inhibitor). Immunoblot analysis was performed using 10–30 μg whole cell lysates. After SDS-PAGE, proteins were transferred to a nitrocellulose membrane, blocked with 5% not-fat skimmed milk in TBS-Tween (0.2%) and immunostained with specific antibodies diluted in TBS-Tween supplemented with 5% milk. FLAG complexes were immunoprecipitated from RNase A (50 μg/ml) treated HEK293 cell lysates using M2 anti-FLAG magnetic beads (Sigma). The beads were washed 3x with lysis buffer and protein complexes were eluted with SDS-sample buffer.

### *In vitro* transcription, *in vitro* splicing and RNP immunoprecipitation

*In vitro* transcription and *in vitro* splicing experiments were performed as previously described (25). In brief, capped transcripts were generated by run-off transcription with SP6 polymerase in the presence of m7GpppG cap analog (Promega) and α-^32^P-GTP. *In vitro* splicing reactions were carried out in HeLa nuclear extracts (CIL Biotech, CC-01–20–50) supplemented with whole cell extracts expressing FLAG-tagged proteins. RNP immunoprecipitations were performed with red anti-FLAG affinity gel (Sigma) in EJC buffer (20 mM HEPES-KOH (pH 7.9)), 200 mM NaCl, 2 mM MgCl_2_, 0.2% Triton-X100, 0.1% Nonidet-P40, 0.05% sodium deoxocholic acid). RNA was recovered with Isol-RNA lysis reagent (5 Prime) and resolved by denaturing PAGE. Signals were quantified on a phosphorimager.

To monitor the assembly of protein complexes at position −24 nt on the mRNA, *in vitro* splicing reactions were combined with oligonucleotide-directed RNase H digestion of the spliced mRNA. After splicing of ^32^P-body-labeled MINX mRNA in HeLa nuclear extracts, a DNA-oligonucleotide (2 μM) complementary to position −24 on the mRNA was added and the reaction incubated for 20 min at 30°C. Subsequently, the RNA was recovered with Isol-RNA lysis reagent and resolved by denaturing PAGE as described before.

### Antibodies

The antibodies against CWC22, FLAG and tubulin were from Sigma. The eIF4A3 polyclonal antibody (rabbit) was raised against an N-terminal peptide of eIF4A3 by GenScript.

### RNA-seq

RNA-seq analysis was carried out on HeLa cells transfected with siRNAs targeting endogenous CWC22, eIF4A3 or the negative control luciferase 48 h prior to harvesting. Two biological replicates were analyzed for each sample. Total RNA was extracted with Isol-RNA lysis reagent (5 Prime) and 1 μg of RNA was used as input material for library preparation. Ribosomal depletion and strand specific library preparation was carried out with the TruSeq® Stranded Total RNA LT (with Ribo-Zero™ Human/Mouse/Rat) according to the manufacturer's instructions. After validation (Agilent 2200 TapeStation) and quantification (Invitrogen Qubit System) all six transcriptome libraries were pooled. The pool was quantified using the Peqlab KAPA Library Quantification Kit and the Applied Biosystems 7900HT Sequence Detection System and loaded on two runs of Illumina Miseq sequencers with a 2×75bp v3 protocol. The analysis produced 1.4–1.5 Gb/sample, corresponding to 8.8–9.8 M reads/sample. Basic read quality check was carried out using FastQC showing >94% of Q30 bases (PF).

### RNA-seq data analysis

RNA-seq data analysis was performed with the Tuxedo software suite: Tophat (release 2.0.13, PMID: 23618408), Cuffdiff (release 2.2.1, PMID: 23222703), cummeRbund (PMID: 22383036) and additional custom scripts. Briefly, trimmed paired-end reads were aligned to the human reference genome and transcriptome (EnsEMBL release 75) allowing for two mismatches. We performed the differential expression analysis on the subset of protein-coding genes applying a *q*-value cutoff of 0.05. GO term enrichment analysis (topGo, PMID: 16606683) as described in the manual using the subset of expressed protein-coding genes as background set. All categories with a *p*-value < 0.05 were retained and a subset was visualized with a custom plotting routine showing enrichment *p*-value, expression fold changes and number of genes simultaneously.

Global estimates of transcript abundance changes over all EnsEMBL biotypes (coding, ncRNA…) were computed from the Cuffdiff isoform-specific FPKM estimates and plotted as a heatmap.

The proportion of intronic reads per sample and gene was determined as follows: all overlapping exons of a given gene locus were merged into super-exons and all 5′ ends of mapped short reads were counted for the whole locus and the union of super-exons. Read counts were obtained with the bedtools software (PMID: 25199790). The proportion of intronic reads is then calculated as 1-(#exonic reads/# gene locus reads). We only consider gene loci with a minimal sum of 100 intronic reads over all conditions. As the exon to intron sizes may be different for every gene, we normalized the calculated proportions by setting the highest ratio across all six samples to 1.

RNA-seq data generated in this study has been uploaded to the NCBI sequence read archive (SRA) with the accession number SRP056271.

### Statistics

*P*-values in Supplementary Figure S3 were calculated with student's *t*-test using GraphPad Prism.

## RESULTS

### CWC22 interacts with early spliceosomal complexes *in vitro*

**Figure 1.**
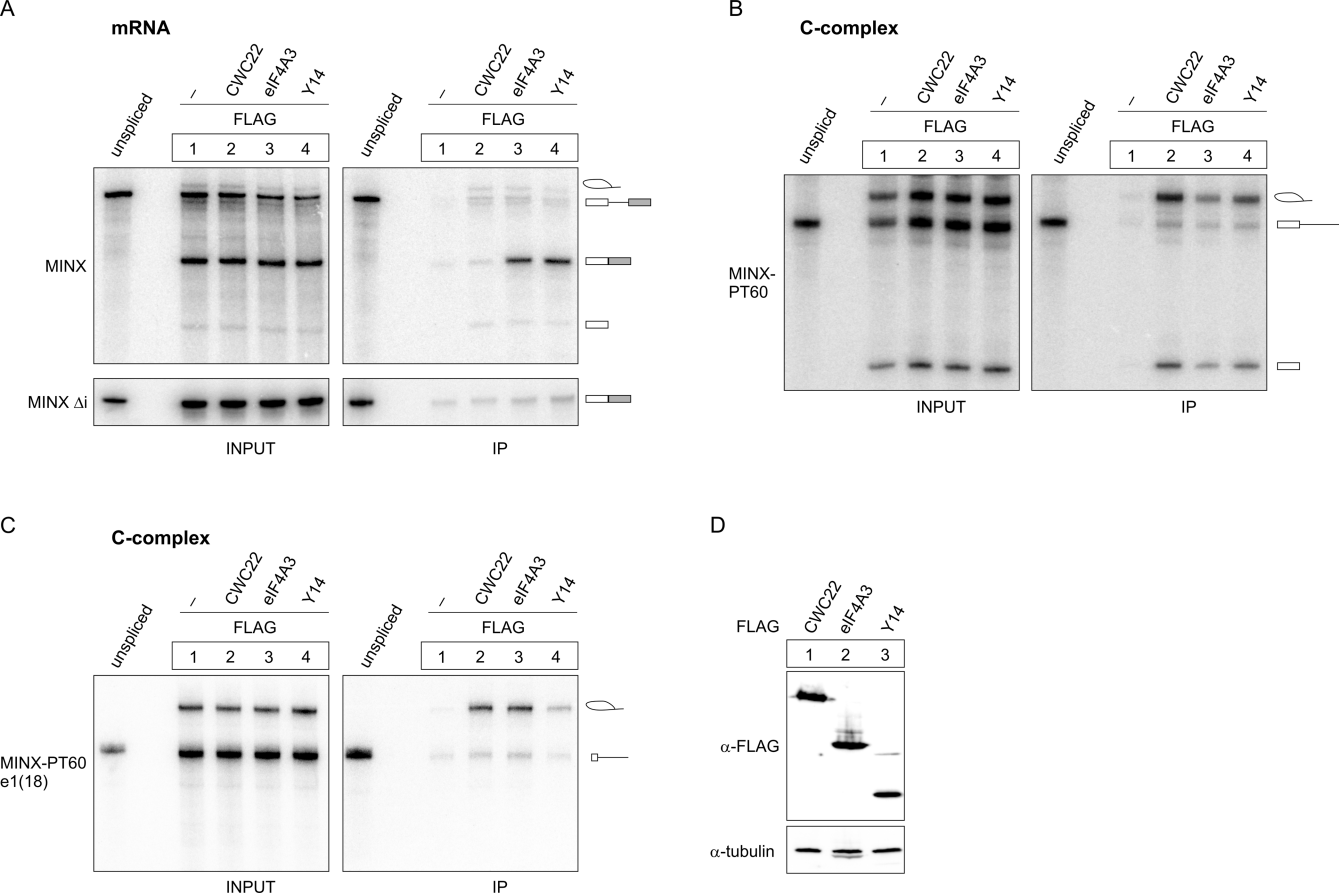
CWC22, eIF4A3 and Y14 differentially interact with spliced mRNA. (**A**–**C**) Splicing of ^32^P-body-labeled MINX (A, top panel), MINX Δi (A, bottom panel), MINX-PT60 (B) or MINX-PT60/e1(18) (C) pre-mRNA in the presence of FLAG-CWC22, FLAG-eIF4A3, FLAG-Y14 or unfused FLAG as a negative control. FLAG-containing mRNPs were immunoprecipitated and the extracted RNA was resolved by denaturing PAGE. 10% of the splicing reaction was loaded as input. Schemes on the right site of the panels depict the splicing products. (**D**) Expression of FLAG-tagged proteins in HEK293 cell extracts was detected by immunostaining with a FLAG-specific antibody. Tubulin served as a loading control.

Mass spectrometric analyses identified CWC22 as an abundant component of the activated pre-catalytic spliceosome (B^act^) and spliceosomal C-complex (26,27), but the molecular details of the spliceosomal association of CWC22 remain vague. We therefore used an *in vitro* system to study the association of CWC22 with spliced mRNA. This assay is based on the splicing of *in vitro* transcribed, radiolabeled pre-mRNAs in HeLa nuclear extracts (24,25). The use of different splice substrates allows the discrimination between splicing-dependent and independent interaction with RNA, as well as the analysis of distinct spliceosomal sub-complexes. In order to compare the mRNA association of CWC22 and the EJC, we supplemented splicing reactions of MINX pre-mRNA with whole cell extracts from HEK293 cells expressing either FLAG-tagged CWC22 or the EJC proteins FLAG-eIF4A3 and FLAG-Y14 (Figure 1A, D). Unfused FLAG served as a negative control. Subsequent FLAG-immunoprecipitation confirmed that FLAG-eIF4A3 and FLAG-Y14 selectively co-precipitated the fully spliced mRNA, indicating that the proteins had been successfully incorporated into splicing-dependent EJCs (Figure 1A, lanes 3 and 4). In contrast, FLAG-CWC22 co-precipitated neither unspliced pre-mRNA nor spliced mRNA, suggesting that the protein is no component of the post-splicing mRNP (Figure 1A, lane 2). This observation is in line with our previous experiments, which showed that CWC22 interacts with eIF4A3, but not with the fully assembled EJC (20). Intronless MINX Δi mRNA confirmed the specificity of the co-IP experiment (Figure 1A, bottom panel). Of note, splicing of MINX pre-mRNA was very efficient and no splicing intermediates could be detected with this experimental setup (Figure 1A). To specifically monitor the interaction of FLAG-CWC22 with splicing intermediates, we performed splicing reactions with MINX-PT60, a pre- mRNA substrate with an elongated polypyrimidine tract (PT60), which lacks a 3′ exon. Due to the missing 3′ splice acceptor site, splicing of MINX-PT60 is stalled after intron-lariat formation in the C-complex spliceosome. As previously shown (24), both FLAG-eIF4A3 and FLAG-Y14 precipitated splicing intermediates (intron-lariat, 5′ exon), indicating that they interact with the spliceosome prior to exon ligation (Figure 1B, lanes 3 and 4). Importantly, FLAG-CWC22 precipitated comparable amounts of splicing intermediates, characterizing it as a component of the spliceosomal C-complex (Figure 1B, lane 2). Moreover, all three proteins precipitated small amounts of unspliced pre-mRNA, which might indicate that they interact with the spliceosome prior to the first catalytic step of splicing (Figure 1B, lanes 2–4).

Similar results were obtained with an AdML (Adenovirus Major Late) splicing substrate, demonstrating that the interactions do not depend on the RNA species (Supplementary Figure S1). Based on these findings, we designed modified splicing experiments to investigate the association of FLAG-CWC22 with the spliceosome in more detail.

### The spliceosomal interaction of CWC22 is independent of the EJC binding site

First, we studied if the mRNA substrate influences the association of FLAG-CWC22 with the spliceosome. Since EJCs are deposited at a fixed distance 20–24 nt upstream of exon–exon junctions (1), shortening the 5′ exon to a length of less than 20 nt prevents EJC assembly (24). Notably, a short 5′ exon also impairs the association of Y14 with early splicing intermediates, whereas eIF4A3 interacts with the spliceosome in the absence of the EJC binding site in the mRNA (24). To test if the spliceosomal association of FLAG-CWC22 requires the EJC binding site, we generated a MINX-PT60 transcript with a shortened 5′ exon (18 nt). This pre-mRNA, which we termed MINX-PT60/e1(18), underwent the first catalytic step of splicing in HeLa nuclear extracts, albeit with a lower efficiency than MINX-PT60 (Figure 1C). We then analyzed the ability of FLAG-CWC22, FLAG-eIF4A3 and FLAG-Y14 to precipitate the splicing intermediates generated from MINX-PT60/e1(18) (Figure 1C). As expected, FLAG-Y14 precipitated less splicing intermediates generated from MINX-PT60/e1(18) than from MINX-PT60, whereas FLAG-eIF4A3 precipitated similar amounts of MINX-PT60 and MINX-PT60/e1(18) intron-lariat RNA (Figure 1C). Interestingly, FLAG-CWC22 also precipitated equal amounts of MINX-PT60 and MINX-PT60/e1(18) splicing intermediates, demonstrating that the spliceosomal association of FLAG-CWC22 occurs independently of the EJC binding site on the mRNA (Figure 1C).

### CWC22 and eIF4A3 sequentially bind to the spliceosome

**Figure 2.**
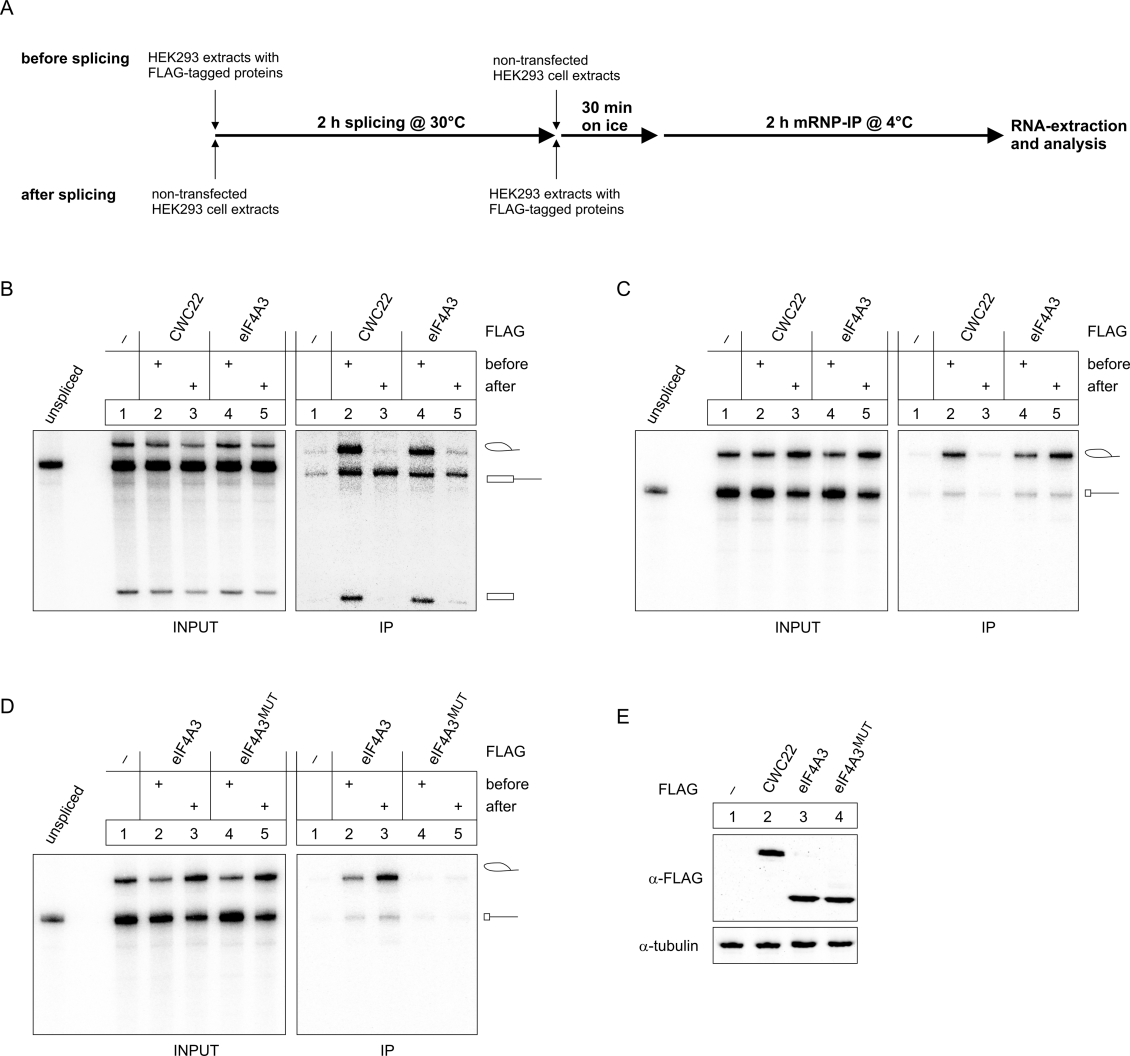
CWC22 and eIF4A3 are sequentially recruited to the spliceosome. (**A**) Scheme of the splicing setup used in (B–D). (**B**–**D**) *In vitro* splicing and mRNP immunoprecipitation of MINX-PT60 (B) or MINX-PT60/e1(18) (C and D) pre-mRNA was performed as described in Figure 1. HEK293 extracts containing the indicated FLAG-tagged proteins or unfused FLAG as negative control were added either before or after the splicing reaction was completed. (D) Expression of FLAG-tagged proteins in HEK293 cell extracts was detected by immunostaining with a FLAG-specific antibody. Tubulin served as a loading control.

We next sought to delineate the process, by which CWC22 and eIF4A3 are recruited to the spliceosome. To this end, we performed splicing reactions, to which we added cell extracts containing FLAG-CWC22 or FLAG-eIF4A3 either before or after splicing was completed (Figure 2A, E). When MINX-PT60 was used as a splicing substrate, FLAG-CWC22 and FLAG-eIF4A3 precipitated splicing intermediates only when the proteins were present at the beginning of the splicing reaction, which suggests that no substantial incorporation of these proteins into spliceosomes takes places after the first catalytic step of splicing (Figure 2B). In contrast, unspliced pre-mRNAs were precipitated regardless of when the proteins were added to the splicing reaction (Figure 2B). Previous studies have indicated that, within the spliceosomal C-complex, eIF4A3 forms a stable, RNA-bound pre-EJC together with MAGOH-Y14 (24). Thus, pre-EJC formation may inhibit the dissociation of eIF4A3 from the C-complex spliceosome. To selectively analyze the interaction of FLAG-eIF4A3 with spliceosomal components, and to minimize the contribution of the RNA and pre-EJC formation, the experiment was repeated using the mRNA substrate MINX-PT60/e1(18), which lacks the EJC-binding site and therefore fails to support pre-EJC formation (Figure 2C). Similar to the splicing reaction with MINX-PT60, FLAG-CWC22 precipitated MINX-PT60/e1(18) intron-lariat mRNA only when the protein was added at the beginning of the splicing reaction (Figure 2C, lanes 2 and 3), confirming that CWC22 has to engage the spliceosome at a pre-catalytic stage. In contrast, FLAG-eIF4A3 precipitated equal amounts of MINX-PT60/e1(18) splicing intermediates, regardless of when it was added to the splicing reaction (Figure 2C, lanes 4 and 5), suggesting that, in the absence of pre-EJC formation, the interaction between eIF4A3 and the spliceosome is transient. We were able to confirm these results using AdML-PT60 and AdML-PT60/e1(18) as splicing substrates (Supplementary Figure S2). Of note, a mutant of FLAG-eIF4A3 (D270K/D273K), which is unable to bind CWC22 (20), completely failed to precipitate MINX-PT60/e1(18), demonstrating that the pre-EJC independent interaction between FLAG-eIF4A3 and the spliceosome is mediated by CWC22 (Figure 2D, lanes 4 and 5). Taken together, our data indicate that the initial spliceosomal recruitment of eIF4A3 is mediated via a temporary interaction with CWC22, whereas C-complex formation initiates pre-EJC assembly and thereby locks eIF4A3 on the mRNA.

### The isolated MIF4G domain of CWC22 inhibits splicing-dependent EJC assembly

The interaction between CWC22 and eIF4A3 is mediated via the MIF4G domain (∼residues 145–350) of CWC22 (20,23). A recent crystallographic study revealed how the N-terminal helices of the MIF4G domain anchor the RecA2 domain of eIF4A3, whereas additional weaker contacts between the RecA1 domain of eIF4A3 and C-terminal helices of CWC22-MIF4G determine the relative orientation of the two RecA-like domains within the complex (23). This bipartite mode of interaction holds the DEAD-box helicase eIF4A3 in an inactive conformation, and it has been speculated that the inhibition of ATPase activity through CWC22 promotes EJC assembly by preventing premature binding to mRNA (21,23).

**Figure 3.**
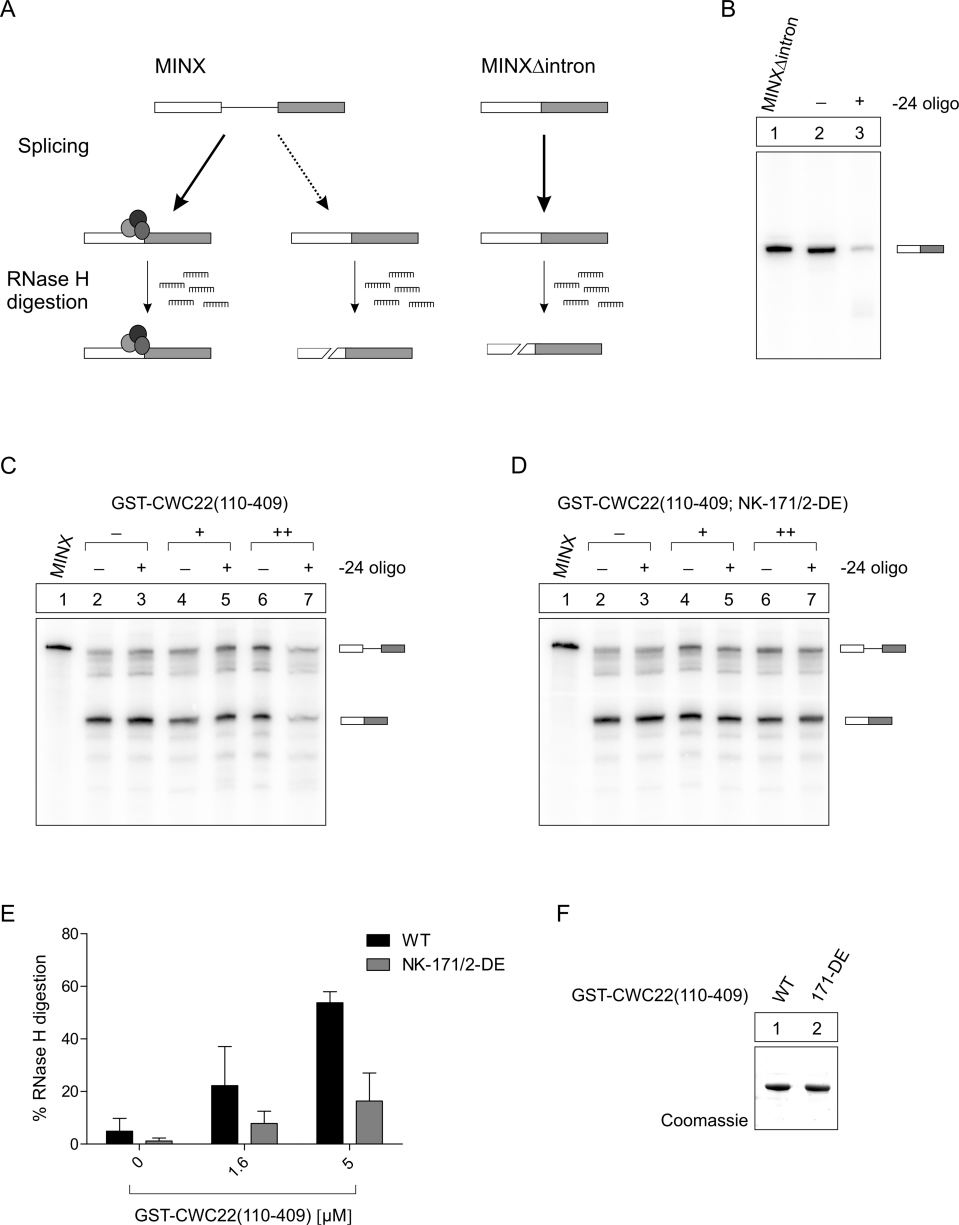
The isolated MIF4G domain of CWC22 inhibits EJC assembly. (**A**) Schematic overview of the experimental approach, depicting how EJCs protect spliced mRNAs from oligonucleotide-directed RNase H digestion. (**B**) Intronless mRNA served as a negative control to determine the maximum RNase H digestion rate. (**C** and **D**) Splicing of ^32^P-body-labeled MINX pre-mRNA in the presence of increasing concentrations (0μM (-), 1.6μM (+), 5μM (++)) of GST-CWC22(110–409) (C) or GST-CWC22(110–409(N171D/K172E) (D); EJC deposition on spliced mRNAs was monitored through oligonucleotide-directed RNase H digestion. (**E**) Quantification of RNase H digestion of spliced mRNAs in (C and D). RNase H digestion of intronless mRNA (B) was set to 100%. Error bars = SD, *n* = 2. (**F**) Coomassie-stained gel showing recombinant proteins added to splicing reactions in (C and D).

To test whether the MIF4G domain alone is sufficient to support splicing-dependent EJC assembly, we supplemented *in vitro* splicing reactions of MINX pre-mRNA with increasing amounts (0μM, 1.6μM and 5μM) of recombinant GST-tagged CWC22-MIF4G (GST-CWC22(110–409)) (Figure 3). EJC formation on spliced mRNA was assessed through oligonucleotide-directed RNase H digestion, using a DNA-oligonucleotide complementary to the EJC binding site (−24 nt) on the mRNA (Figure 3A). This position is protected from RNase H by the bound EJC after splicing of the RNA (Figure 3A, 3C lanes 1 and 2), but is cleaved by RNase H in unprotected MINX RNA (Figure 3A, B). Interestingly, addition of GST-CWC22(110–409) to splicing reactions increased the sensitivity to RNase H digestion of the spliced mRNA in a concentration-dependent manner, suggesting that GST-CWC22(110–409) inhibits the splicing-dependent assembly of EJCs (Figure 3C, E, F). In contrast, only minimal deprotection of spliced mRNA was observed when the splicing reaction was supplemented with an eIF4A3-binding deficient mutant of CWC22 (CWC22(110–409(N171D/K172E)), demonstrating that the direct interaction between eIF4A3 and CWC22-MIF4G was required to reduce EJC assembly (20) (Figure 3D–F). Hence, the isolated MIF4G domain of CWC22 inhibits the assembly of EJCs during *in vitro* splicing reactions (Figure 3), which is in line with our previous results showing that CWC22-MIF4G impairs the formation of recombinant EJCs *in* vitro (20). Together, these findings suggest that additional domains of CWC22 are required for its function during the splicing-dependent deposition of EJCs.

### Several domains of CWC22 are needed for its efficient spliceosomal association

**Figure 4.**
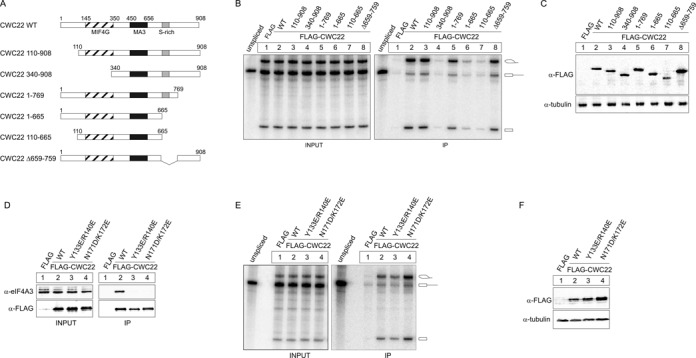
Several domains of CWC22 mediate the eIF4A3-independent interaction of CWC22 with the spliceosome. (**A**) Schematic representation of deletion mutants of CWC22 used in (B). (**B**) *In vitro* splicing and mRNP-immunoprecipitation of MINX-PT60 pre-mRNA in the presence of FLAG-tagged CWC22 deletion mutants was performed as described in Figure 1. (**C**) Expression of FLAG-tagged proteins in HEK293 cell extracts was detected by immunostaining with a FLAG-specific antibody. Tubulin served as a loading control. (**D**) FLAG-immunoprecipitation of CWC22 mutants from RNase A treated HEK293 cell lysates. Co-precipitated endogenous eIF4A3 was detected by immunostaining with a specific antibody. Unfused FLAG served as a negative control. 10% of the cell lysate was loaded as input. (**E**) *In vitro* splicing and mRNP immunoprecipitation of MINX-PT60 pre-mRNA in the presence of the indicated FLAG-tagged CWC22 mutants was performed as described in Figure 1. (**F**) Expression of FLAG-tagged proteins in HEK293 cell extracts was detected by immunostaining with a FLAG-specific antibody. Tubulin served as a loading control.

Next, we aimed to identify domains of CWC22 that mediate the interaction with spliceosomes. To this end, we carried out *in vitro* splicing reactions of MINX-PT60 pre-mRNA or AdML-PT60 pre-mRNA in the presence of different truncated versions of FLAG-CWC22 (Figure 4A). Human CWC22 has three characteristic domains, a MIF4G domain (∼amino acid residues 145–350), an MA3 domain (∼450–656) and an S-domain (∼665–717). The MIF4G domain of CWC22 binds to eIF4A3 (20,23), whereas both MIF4G and MA3 domain are required for pre-mRNA splicing (20). Based on this knowledge, we designed mutants that lacked the N- or C-terminal regions of the protein, in combination with deletions of the MIF4G and the S-domain (Figure 4A). Deleting the N-terminal part of the protein (amino acid residues 1–109) did not influence its interaction with splicing intermediates (Figure 4B, lane 3, Supplementary Figure S3A). In contrast, the spliceosomal interaction was lost when the N-terminus was deleted in combination with the MIF4G domain (Figure 4B, lane 4, Supplementary Figure S3A). Interestingly, stepwise deletion of the C-terminus led to a gradual decrease in the ability to co-precipitate splicing intermediates: Deleting the last 139 C-terminal amino acids of FLAG-CWC22 (1–769) caused a slight reduction (Figure 4B, lane 5, Supplementary Figure S3A), which was further aggravated upon deletion of an additional 104 amino acids (1–665), including the S-domain (Figure 4B, lane 6, Supplementary Figure S3A). Of note, an internal deletion of the S-domain caused only a slight decrease in the RNA precipitation (Figure 4B, lane 8, Supplementary Figure S3A). This gradual effect suggests that several regions within the C-terminus of CWC22 contribute to the interaction with spliceosomal components.

Importantly, a FLAG-tagged CWC22 protein comprising of MIF4G and MA3 domain (110–665) displayed a weak, but consistent affinity for splicing intermediates (Figure 4B, lane 7, Supplementary Figure S3A, S3B). We have previously shown that this piece of CWC22 restores pre-mRNA splicing in CWC22-depleted cells (20). This suggests that, although the interaction with spliceosomal components is strongly enhanced by the C-terminal domain, the conserved core of CWC22 is sufficient to partially sustain binding to the spliceosome.

### CWC22 interacts with the C-complex of the spliceosome independently of eIF4A3

Our *in vitro* splicing experiments showed that the MIF4G domain of CWC22 is obligatory to precipitate MINX-PT60 and AdML-PT60 splicing intermediates (Figure 4B, lane 4). Since the MIF4G domain also mediates binding to eIF4A3, we asked whether the interaction with eIF4A3 is required for the spliceosomal recruitment of CWC22. To this end, we performed *in vitro* splicing experiments with FLAG-CWC22 variants, in which the eIF4A3-binding site was mutated (Figure 4D-3F). Mutating Asn171 and Lys172 of CWC22 (N171D/K172E) completely abolishes the binding to eIF4A3 (20). Additionally a Y133E/R140E mutant was recently reported to possess a 100-fold lower affinity for eIF4A3 *in vitro* (23). In co-immunoprecipitation experiments, this reduced affinity resulted in a complete loss of detectable interaction with endogenous eIF4A3, comparable to that observed for CWC22 (N171D/K172E) (Figure 4D). Interestingly, none of the eIF4A3-binding deficient FLAG-CWC22 mutants showed a reduced ability to precipitate splicing intermediates generated from MINX-PT60 or AdML-PT60 pre-mRNA (Figure 4E, Supplementary Figure S3C). Indeed, FLAG-CWC22 (N171D/K172E) precipitated slightly more splicing intermediates than the wild type protein, possibly due to a higher expression of the mutant protein in HEK293 cell extracts (Figure 4F). Taken together, these results demonstrate that the interaction with eIF4A3 is dispensable for the recruitment of CWC22 to the splicing machinery and suggests that the MIF4G domain is involved in eIF4A3-independent binding to the human spliceosome.

### The interaction with eIF4A3 is dispensable for the splicing function of CWC22

**Figure 5.**
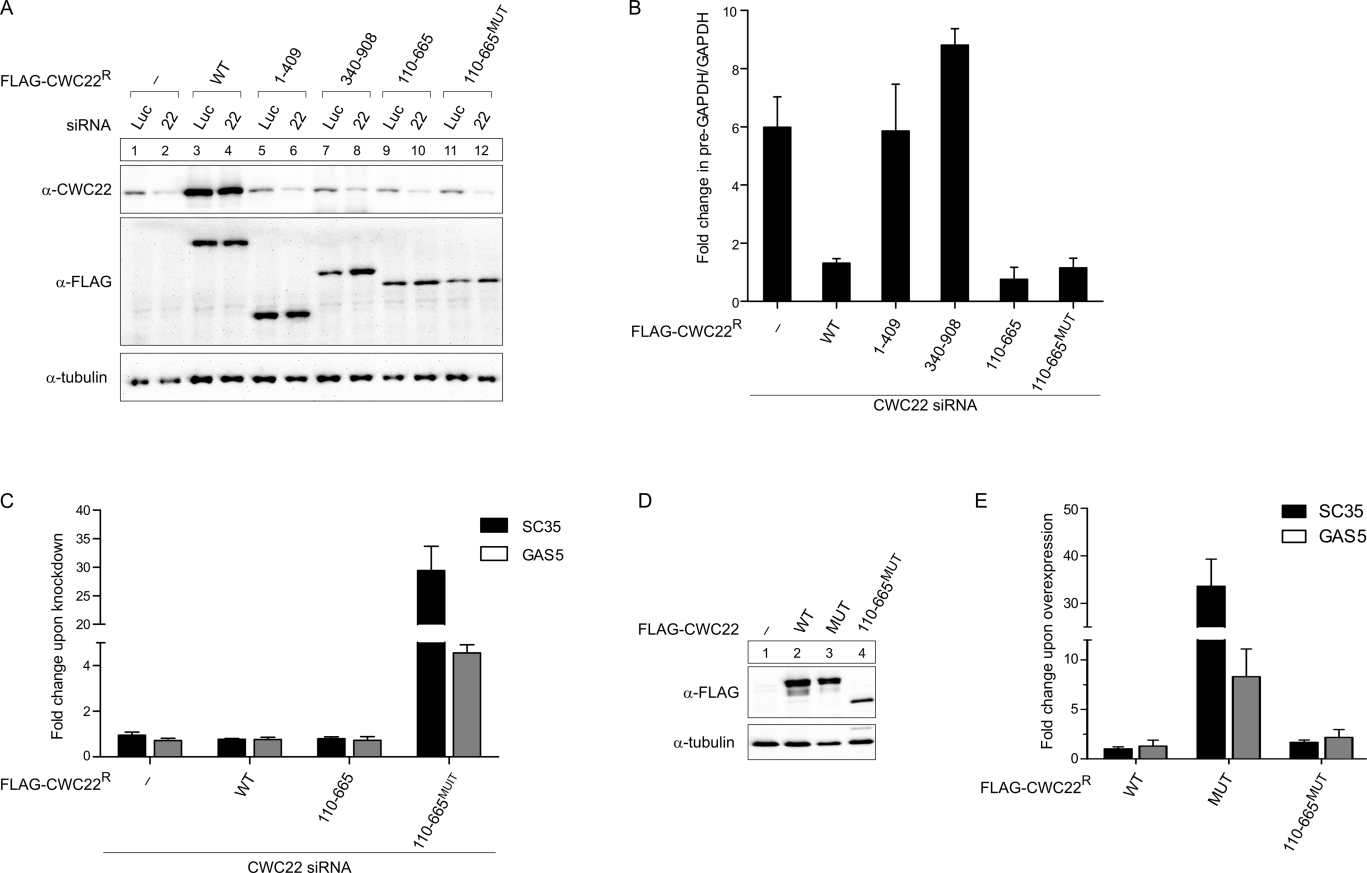
Pre-mRNA splicing and EJC assembly can be experimentally uncoupled. (**A**) HEK293 Flp-In cells expressing FLAG-tagged CWC22 or the indicated deletion mutants were transfected with siRNAs targeting CWC22 or luciferase as a negative control. Unfused FLAG served as a negative control. Expression of endogenous CWC22 (top panel) and FLAG-tagged CWC22 (middle panel) was detected by immunostaining with specific antibodies. Tubulin (bottom panel) served as a loading control. (**B**) qPCR analysis determining the fold change in the GAPDH pre-mRNA/mRNA ratio upon CWC22-depletion in HEK293 Flp-In cells expressing the indicated CWC22 proteins or unfused FLAG as a negative control. Error bars = SD, *n* = 3. (**C**) qPCR analysis determining the fold change in SC35 (1.7 kb splice product) and GAS5 mRNA levels upon CWC22-depletion in HEK293 Flp-In cells expressing the indicated CWC22 proteins. Expression levels were normalized to the housekeeping gene TATA-box binding protein (TBP). Error bars = SD, *n* = 3. (**D**) Expression of FLAG-tagged CWC22 proteins upon doxycycline-treatment of HEK293 Flp-In cells was detected by immunostaining with a FLAG-specific antibody. Tubulin served as a loading control. (**E**) qPCR analysis determining the fold change in SC35 and GAS5 mRNA levels (normalized to TBP) upon overexpression of the indicated CWC22 proteins in HEK293 Flp-In cells. Error bars = SD, *n* = 3.

Recent studies have shown that the EJC proteins eIF4A3, MAGOH and Y14 regulate splicing of a subset of transcripts in *Drosophila* and human cells (13–17). CWC22 has been speculated to be involved in the splicing function of EJC proteins, because it is a direct interaction partner of eIF4A3 and an essential splicing factor in human cells (20,22). However, a link between the splicing function of CWC22 and its role during EJC assembly has not been analyzed experimentally. To systematically study these two functions of CWC22, we established a cellular complementation assay and generated stable HEK293 Flp-In cell lines expressing doxycycline-inducible, siRNA-resistant FLAG-tagged CWC22 constructs (Figure 5A). We then depleted endogenous CWC22 using specific siRNAs (Figure 5A) and employed quantitative RT-PCR (qPCR) to analyze the ability of different FLAG-CWC22 constructs to rescue the splicing defect (Figure 5B). So far, this had only been studied with transfected reporter mRNAs (20). The use of qPCR allowed us to selectively analyze the splicing of endogenous transcripts. As shown by a ∼6-fold increase in GAPDH pre-mRNA relative to GAPDH mRNA levels, CWC22-depletion led to a severe splicing defect, which could be rescued by inducing the expression of siRNA-resistant FLAG-CWC22 (Figure 5B). Importantly, neither the N-terminal part of CWC22 (1–409), nor the C-terminal part (340–908), was able to restore splicing in CWC22-depleted cells (Figure 5B). These results are in line with our previous experiments, showing that both MIF4G and MA3 domain are required for pre-mRNA splicing in human cells (20). Accordingly, the central segment (110–665) of CWC22, comprising of MIF4G and MA3 domain, was sufficient to maintain splicing in CWC22-depleted cells (Figure 5B). In order to determine whether the interaction with eIF4A3 is required for the splicing function of CWC22, we expressed FLAG-CWC22 with a mutated (N171D/K172E) eIF4A3-binding site (FLAG-CWC22^MUT^) in HEK293 Flp-In cells. Surprisingly, doxycycline induction of the mutant transgene strongly impaired cell viability. Depleting endogenous CWC22 in FLAG-CWC22^MUT^-expressing cells further aggravated the effect and thereby prevented the analysis of pre-mRNA splicing by qPCR. To avoid the adverse effects associated with the expression of FLAG-CWC22^MUT^, we used FLAG-CWC22 (110–665)^MUT^ to carry out the complementation experiment. Interestingly, FLAG-CWC22 (110–665)^MUT^ supported pre-mRNA splicing with a similar efficiency as the wild type protein (Figure 5B), demonstrating that the interaction between CWC22 and eIF4A3 is dispensable for the general splicing function of CWC22.

Next, we analyzed, which CWC22 variants supported EJC assembly in CWC22-depleted cells (Figure 5C). Due to the lack of assays to quantify the number of EJCs in living cells, we measured the mRNA level of the endogenous NMD targets SC35 (1.7 kb splice product) and GAS5 by qPCR. The 1.7 kb transcript of SC35 is part of an autoregulatory feedback loop that controls SC35 expression through non-productive alternative splicing, whereas the snoRNA-encoding pseudogene GAS5 contains several PTCs (28,29). Due to their aberrant architecture, both transcripts are rapidly degraded via EJC-dependent NMD. As expected, depletion of the essential EJC protein Y14 impaired NMD and led to a strong upregulation of SC35 and GAS5 mRNA levels (Supplementary Figure S4). Interestingly, the knockdown of CWC22 did not cause an increase in SC35 and GAS5 expression levels, which is most likely due to impaired pre-mRNA splicing in CWC22-depleted cells. However, when the depletion of endogenous CWC22 was complemented with a splicing-competent, but eIF4A3-binding deficient mutant of FLAG-CWC22 (110–665^MUT^), a strong upregulation of both SC35 and GAS5 was observed (Figure 5C). In contrast, EJC assembly was completely rescued by overexpressing siRNA-resistant wild type FLAG-CWC22. Interestingly, the central part of the CWC22 (110–665) was equally capable to maintain NMD of both mRNAs, demonstrating that MIF4G and MA3 domain are sufficient to promote EJC assembly in CWC22-depleted cells (Figure 5C).

Taken together, our results demonstrate that the core of CWC22, consisting of MIF4G and MA3 domain, is sufficient for both pre-mRNA splicing and EJC assembly in CWC22-depleted cells. Although the interaction with eIF4A3 is crucial for EJC assembly, it is not required for the splicing function of CWC22. This suggests that the two functions of CWC22 are not directly linked and can be experimentally uncoupled.

### Uncoupling of pre-mRNA splicing and EJC assembly with an eIF4A3-binding deficient CWC22 mutant

While establishing the cellular complementation assay, we observed that FLAG-CWC22^MUT^ expression strongly impairs cell viability. The roles of CWC22 in pre-mRNA splicing and EJC assembly prompted us to test which of these processes is impaired in the presence of FLAG-CWC22^MUT^. To this end, we expressed FLAG-CWC22^WT^, FLAG-CWC22^MUT^ and FLAG-CWC22(110–665)^MUT^ in HEK293 Flp-In cells (Figure 5D) and analyzed pre-mRNA splicing and EJC assembly by qPCR. Notably, expression of FLAG-CWC22^MUT^ only mildly inhibited splicing (increase of pre-GAPDH/GAPDH by a factor of 2), which is unlikely to be the reason for the strongly reduced cell viability (Supplementary Figure S5). Expression of FLAG-CWC22^WT^ or FLAG-CWC22 (110–665)^MUT^ did not alter the efficiency of pre-mRNA splicing (Supplementary Figure S5). In contrast to splicing, a drastic increase of SC35 and GAS5 mRNA levels was observed when FLAG-CWC22^MUT^ was overexpressed, suggesting that FLAG-CWC22^MUT^ perturbs EJC assembly in the presence of endogenous CWC22 (Figure 5E). Interestingly, neither FLAG-CWC22^WT^, nor FLAG-CWC22 (110–665)^MUT^ overexpression caused any upregulation of endogenous NMD targets (Figure 5E). This demonstrates that the central segment of CWC22, albeit being able to rescue EJC assembly in CWC22-depleted cells, is unable to exert a dominant negative effect on EJC assembly in the presence of endogenous CWC22. The inability of FLAG-CWC22(110–665)^MUT^ to disturb EJC assembly in living cells thus can be explained by the reduced spliceosomal affinity of CWC22 (110–665) observed *in vitro* (Figure 4B).

### CWC22 depletion causes widespread intron retention and downregulation of components of the gene expression machinery

To date, the splicing function of CWC22 has only been studied for a few model substrates (20,22). We therefore aimed to unambiguously define the role of CWC22 during pre-mRNA splicing by high-throughput sequencing. To this end, RNA-seq analysis was performed on RNA from HeLa cells in which CWC22 or eIF4A3 were depleted with specific siRNAs (Supplementary Figure S6A). In total, the RNA-seq analysis yielded 8.8–9.8 M reads/sample and showed a high degree of correlation between both biological replicates (Supplementary Figure S6B).

**Figure 6.**
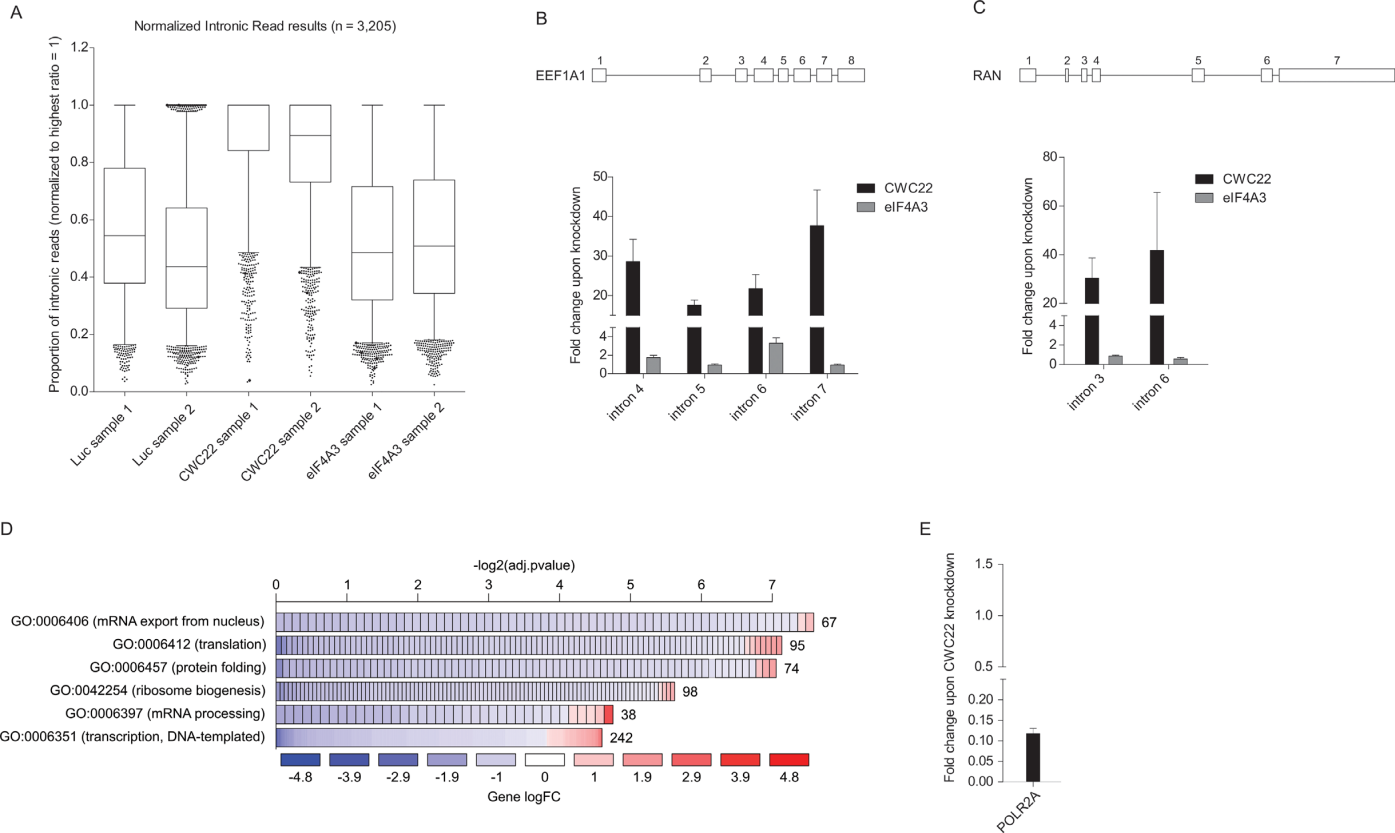
RNA-seq analysis reveals widespread intron retention and downregulation of components of the gene expression machinery upon CWC22 depletion. (**A**) Proportion of intronic reads in RNA from cells treated with the indicated siRNAs as determined by RNA-seq. The highest ratio of intronic/exonic reads per gene was set to 1. Wiskers depict percentile 5–95. (**B** and **C**) qPCR analysis determining the fold change in the pre-mRNA/mRNA ratio of EEF1A1 (B) or RAN (C) upon CWC22-depletion. Schemes above the graphs represent the exon-intron structure of the transcript. Error bars = SD, *n* = 3. (**D**) Enrichment of gene onthology (GO) terms in the group of regulated genes upon CWC22 depletion. The width of the bars represents the significance (–log2(adjusted *p*-value)) of the respective GO term enrichment. Colors depict the log fold change (log FC) of individual genes within the GO category and numbers behind the bars correspond to the number of genes within the GO category. A more detailed GO enrichment analysis is shown in Supplementary Figure S6. (**E**) qPCR analysis determining the fold change in POL2RA mRNA levels upon CWC22 depletion (normalized to EEF1A1 mRNA) compared to control samples. Error bars = SD, *n* = 3.

When transcript abundance changes in RNA from CWC22-depleted cells were estimated globally, we found the most prevalent changes in the group of retained introns (Supplementary Figure S6C). Furthermore, we detected a strong increase in reads mapping to introns, indicating that pre-mRNA splicing is impaired in the absence of CWC22 (Figure 6A, Supplementary Table S1). In contrast, no increase of intronic reads was observed in eIF4A3-knockdown samples, demonstrating that eIF4A3 is dispensable for the splicing of most transcripts (Figure 6A). Previous studies have shown that several EJC proteins, including eIF4A3, are involved in the (alternative) splicing of specific genes in both *Drosophila* and human cells (13,14,16,17,30). In line with these previous findings, a reduced number of reads mapped to KPNA1 alternative exon 11 in both eIF4A3-knockdown samples (Supplementary Figure S6D) (30). In contrast, no KPNA1 exon 11 skipping was observed in CWC22-knockdown samples, indicating that CWC22 is required for general pre-mRNA splicing, but has only little effect on the regulation of alternative splicing.

According to the high-throughput analysis, splicing of some introns appeared to be unaffected by the knockdown of CWC22. While it is conceivable that a small number of introns may indeed be efficiently spliced with reduced CWC22 levels, we speculated that a lack of mappable reads was the main reason for apparent CWC22-independent introns. This hypothesis was supported by our qPCR analysis, which showed comparable inhibition of splicing of several individual introns of the same transcript (Figure 6B and C). Specifically, we found that intron retention in EEF1A1 and RAN transcripts was increased at least by a factor of 20 (Figure 6B and C).

In addition to a prominent splicing defect, CWC22 depletion caused a global downregulation of genes involved in many gene expression pathways (Supplementary Table S2). Gene ontology analysis revealed a significant overrepresentation of genes involved in transcription, translation, mRNA processing, ribosome biogenesis and mRNA export in the group of downregulated genes (Figure 6D, Supplementary Figure S6E, Supplementary Table S3). A particular strong decrease was observed for the largest subunit of RNA polymerase II (POLR2A). This result was confirmed by qPCR, which showed a 10-fold reduction in mRNA levels compared to control samples (Figure 6E). Importantly, this effect was not observed upon eIF4A3 depletion, suggesting that the downregulation of gene expression pathways represents a specific response to the depletion of the splicing factor CWC22.

Taken together, RNA-seq analysis revealed that CWC22 is a global splicing factor involved in the splicing of most—if not all—pre-mRNAs. Moreover, CWC22-depletion leads to a strong downregulation of genes involved in gene expression. This might represent a previously unrecognized feedback mechanism to reduce gene expression pathways in response to inefficient splicing.

## DISCUSSION

**Figure 7.**
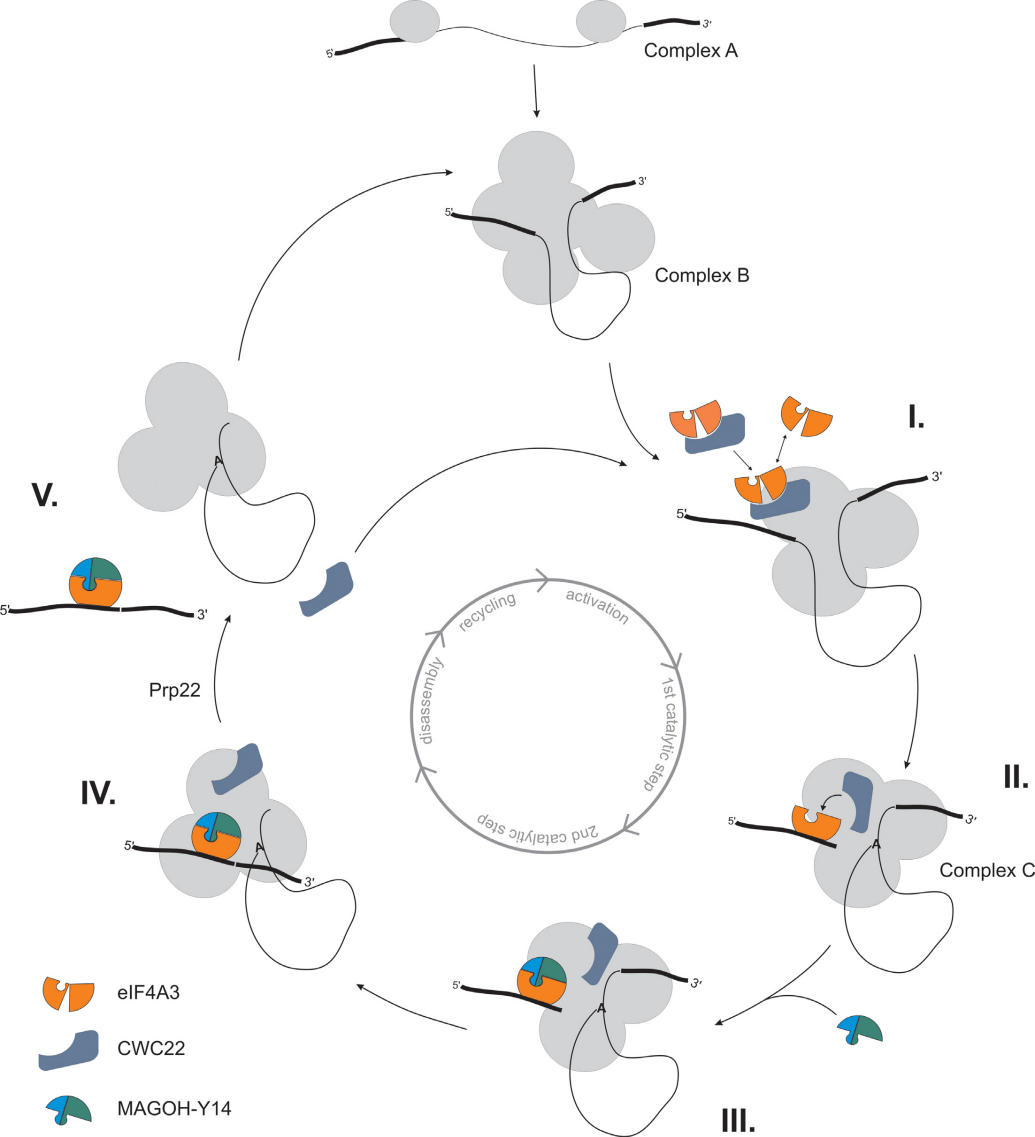
CWC22 guides splicing-dependent EJC assembly. Model of the splicing-dependent eIF4A3-deposition on mRNA through CWC22. Circle in the center of the scheme represents the different steps of pre-mRNA splicing. For details see discussion.

As an essential regulator of both pre-mRNA splicing and EJC assembly, CWC22 plays a pivotal role in the maturation of mRNPs. In this study, we systematically analyzed the spliceosomal function of CWC22 in order to gain a better understanding of the relationship between pre-mRNA splicing and EJC assembly. Using *in vitro* splicing experiments, we uncover the order of events during the spliceosomal recruitment of CWC22 and eIF4A3. Based on these results, we propose a detailed model of how EJC assembly is mediated by CWC22 (Figure 7): CWC22 and eIF4A3 are recruited to the spliceosome prior to the first catalytic step of splicing (as part of the B-complex spliceosome). While CWC22 associates stably with components of the early spliceosome, eIF4A3 appears to be mobile and its initial spliceosomal interaction (via CWC22) is transient *(I.)*. Our data do not provide evidence that the composite surface of a CWC22/eIF4A3 heterodimer is required for the spliceosomal interaction of either eIF4A3 or CWC22. In fact eIF4A3 is able to bind to CWC22, which has been incorporated into the spliceosome. Stable binding of eIF4A3 occurs for the first time when it interacts with the mRNA during spliceosomal activation or the first catalytic step of splicing *(II.)* (24). Subsequently, durable RNA binding is achieved through interaction with MAGOH-Y14 *(III.)*. Since binding of eIF4A3 to MAGOH-Y14 and CWC22 is mutually exclusive (20,21), this step disrupts binding of CWC22 to eIF4A3 *(IV.)*. It is conceivable that additional factors facilitate the dissociation of eIF4A3 from CWC22 in order to enable mRNA binding. However, the mechanism of this process as well as the factors involved remains elusive. In a recent study, the dissociation of the yeast spliceosome was analyzed in a purified in vitro system. This work revealed that CWC22 is separated from the post-catalytic spliceosome *(V.)*, when the DEAH-box helicase HRH1/Prp22 releases the mature mRNP from the intron-lariat spliceosome (31). We therefore propose that by liberating CWC22 from the post-catalytic spliceosome, HRH1/Prp22 may serve as an important recycling factor for CWC22 and replenish the reservoir of available CWC22. This could explain the observation of an earlier study, which showed that an ATPase-deficient mutant of HRH1/Prp22 (‘LAT’) prevents EJC assembly (32). However, a ‘recycling’ function of Prp22 would only affect EJC assembly if the amounts of CWC22 were limiting, which still needs to be tested. Further studies will be required to fully unravel the mechanisms that govern the spliceosomal association and dissociation of CWC22, and the importance of its efficient recycling.

The dual role of CWC22 in pre-mRNA splicing and EJC assembly has led to the hypothesis that EJC-deposition serves as a checkpoint, which licenses the late steps of splicing (22). However, by uncoupling pre-mRNA splicing and EJC assembly, we could demonstrate that the splicing function of CWC22 does not depend on the binding to eIF4A3. These results are in line with previous experiments, showing that eIF4A3-depleted cell extracts retain full splicing activity (32). Thus, rather than comprising a general control mechanism of constitutive splicing, EJCs might regulate the splicing of transcripts with an unfavorable architecture, as has been recently demonstrated for the piwi mRNA in *Drosophila* (16,17).

A central piece of CWC22, comprising of MIF4G and MA3 domain, rescues pre-mRNA splicing and restores EJC assembly in CWC22-depleted cells, thus forming the minimal functional unit of CWC22. Interestingly however, the C-terminal part of the protein greatly enhanced the interaction with the spliceosome. Moreover, an eIF4A3-binding deficient mutant of CWC22, but not a C-terminally truncated version thereof, impairs EJC assembly even in the presence of endogenous CWC22. Together, these findings indicate that motifs in the C-terminal part of CWC22 regulate its function. In contrast to the highly structured core of CWC22, the C-terminus is an intrinsically unstructured region, comprising of an S-domain (amino acid residues 665–717), followed by a 190 amino acid C-terminal tail, which is rich in basic amino acids. The S-domain is found in all higher eukaryotes, but not in yeast (Supplementary Figure S7). We therefore propose that the S-domain has evolved to regulate spliceosomal functions specifically required in higher eukaryotes, such as alternative splicing or the assembly of splicing-dependent EJCs. Interestingly, disordered regions have been described to contain many important interaction surfaces for both proteins and RNA (33). These interactions seem to be particularly important in mRNA metabolism, as shown by an overrepresentation of intrinsically unstructured regions in proteins of the mRNA-interactome (34). Furthermore, basic disordered tails are often found in transcription factors, were they mediate the interaction with DNA (35). Together, these findings suggest that the C-terminus of CWC22 might strengthen the interaction with the spliceosome by facilitating additional interactions with proteins and/or the RNA. Although the C-terminus clearly enhanced the spliceosomal interaction of CWC22, the central part of CWC22 (110–665) was equally active as the wild type protein in cellular complementation assays. While all experiments described in this study were performed using robust substrate mRNAs under standard laboratory conditions, enhanced spliceosomal binding might only be important under stress conditions or for the processing of specific transcripts. Transcriptome-wide analyses, as well as studies that focus on CWC22 function under stress conditions, could therefore extend our knowledge on the regulatory mechanisms that control the activity of CWC22 and the specific role of its C-terminal domain.

Our high-throughput sequencing analysis of CWC22-depleted cells corroborated that CWC22 functions as a general splicing factor in human cells. Splicing inhibition coincided with a marked downregulation of many mRNAs encoding gene expression factors. A striking example is the strongly reduced expression of the catalytic subunit of the RNA polymerase II. Reduced levels of RNA polymerase II will likely decrease the production of new precursor mRNAs and therefore the amount of introns that need to be spliced by the spliceosome. The shutdown of gene expression in CWC22-depleted cells might thus reduce the competition of pre-RNAs for the limiting amount of active spliceosomes and alleviate the splicing defect. Since a number of human disorders is caused by mutations in core spliceosomal components (36), it has been suggested that pre-mRNAs compete for limiting RNA splicing and processing factors (37). How a disproportionate competition for the splicing machinery is mechanistically detected and what molecular characteristics define competitive splicing substrates will be interesting directions for future research.

The global inhibition of splicing in CWC22 depleted cells was confirmed for several individual introns by qPCR. We therefore speculate that strictly CWC22-dependent splicing of all introns in combination with eIF4A3 binding results in a global deposition of EJCs on spliced exon–exon junctions. This is in line with the finding that the majority of exon junctions carries an EJC (38,39). Since eIF4A3 binding is not required for the splicing reaction itself, reduced levels of eIF4A3 do not interfere with splicing, but only result in a decreased assembly of EJCs on spliced mRNAs. The availability of eIF4A3 during the formation of the spliceosome may therefore determine the differential deposition of EJCs on spliced mRNAs. Future studies will be required to understand how the interaction between eIF4A3 and the spliceosome is regulated.

## SUPPLEMENTARY DATA

Supplementary Data are available at NAR Online.

SUPPLEMENTARY DATA
