# Supplementary material for: CWC22-dependent pre-mRNA splicing and eIF4A3 binding enables global deposition of exon junction complexes
Source: Nucleic Acids Res. 2015 Apr 13;43(9):4687–700. doi: 10.1093/nar/gkv320 (PMC4482076; doi:10.1093/nar/gkv320)
Supplement: SUPPLEMENTARY DATA [file supp_43_9_4687__index.html]

CWC22-dependent pre-mRNA splicing and eIF4A3 binding enables global deposition of exon junction complexes — SUPPLEMENTARY DATA 

# CWC22-dependent pre-mRNA splicing and eIF4A3 binding enables global deposition of exon junction complexes

## SUPPLEMENTARY DATA

**Files in this Data Supplement:**

- SUPPLEMENTARY DATA
- SUPPLEMENTARY DATA
- SUPPLEMENTARY DATA
- SUPPLEMENTARY DATA
