# Supplementary material for: CWC22-dependent pre-mRNA splicing and eIF4A3 binding enables global deposition of exon junction complexes
Source: Nucleic Acids Res. 2015 Apr 13;43(9):4687–700. doi: 10.1093/nar/gkv320 (PMC4482076; doi:10.1093/nar/gkv320)
Supplement: SUPPLEMENTARY DATA [file supp_gkv320_nar-03333-a-2014-File009.pdf]

Supplementary Information

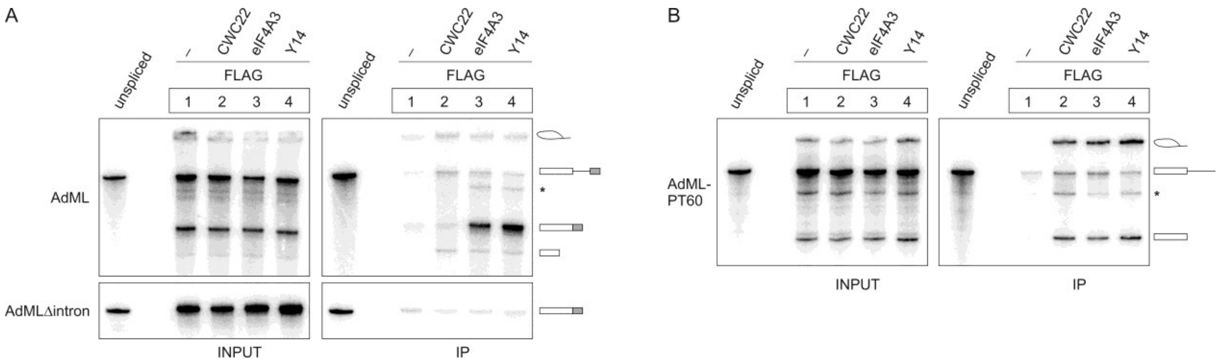

Supplementary Figure 1: CWC22, eIF4A3 and Y14 differentially precipitate AdML pre-mRNA. Splicing of ^{32}P-body-labeled AdML (A, top panel), AdML Δi (A, bottom panel), and AdML-PT60 (B) pre-mRNA in the presence of FLAG-CWC22, FLAG-eIF4A3, FLAG-Y14 or unfused FLAG as a negative control. FLAG-containing mRNPs were immunoprecipitated and the extracted RNA was resolved by denaturing PAGE. 10% of the splicing reaction was loaded as input. Schemes on the right site of the panels depict the splicing products.

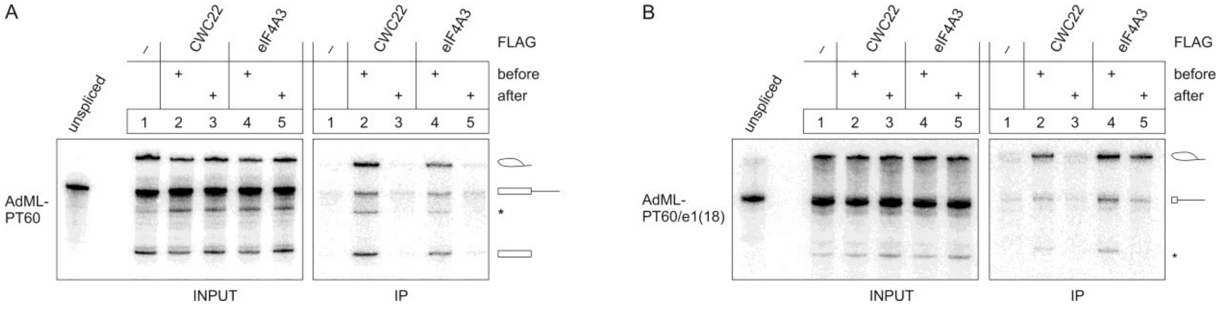

Supplementary Figure 2: Sequential spliceosomal recruitment of CWC22 and eIF4A3 analyzed through splicing of AdML pre-mRNA.

In vitro splicing and mRNP immunoprecipitation of AdML-PT60 (A) or AdML-PT60/e1(18) (B) pre-mRNA was performed as described in Supplementary Figure 1. HEK293 extracts containing the indicated FLAG-tagged proteins or unfused FLAG as negative control were added either before or after the splicing reaction was completed.

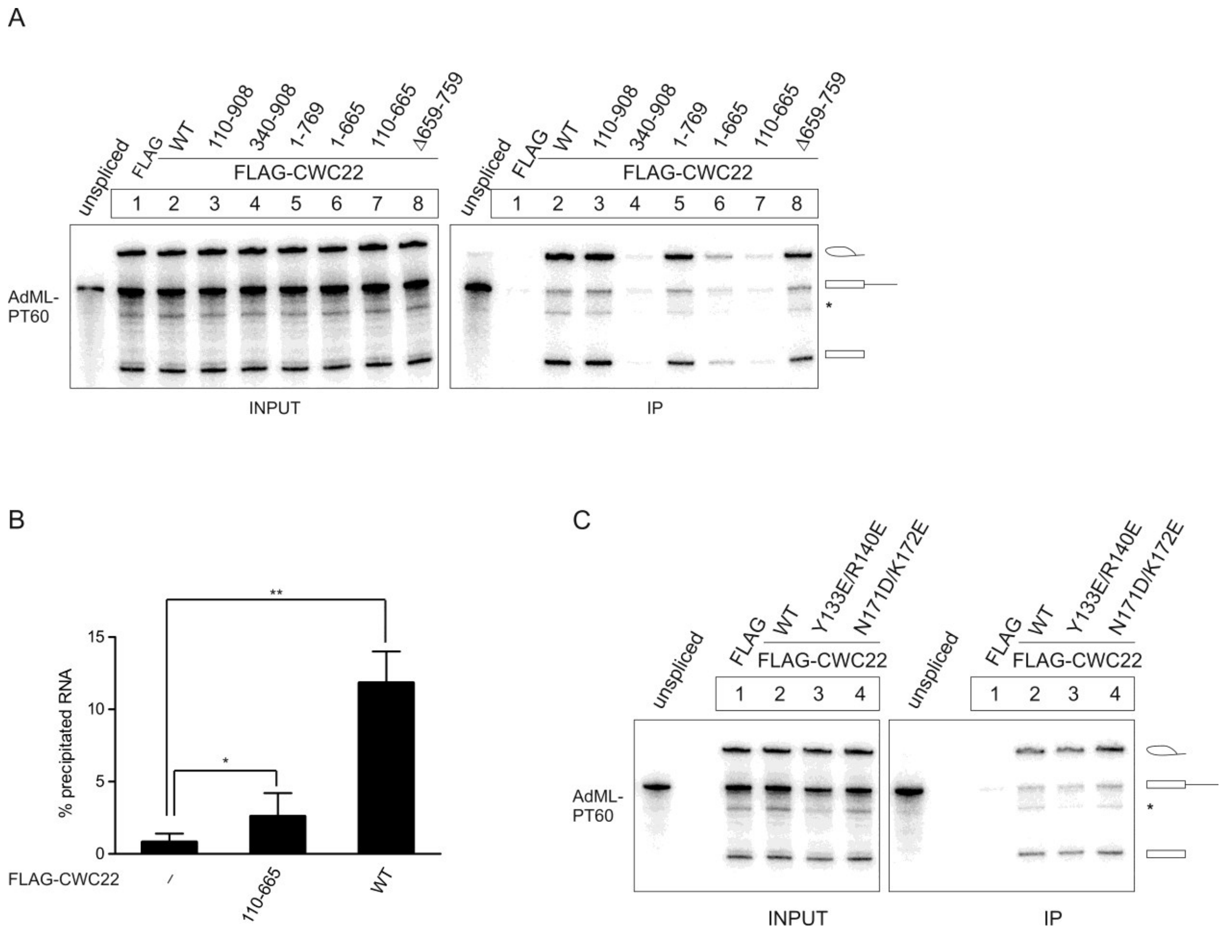

Supplementary Figure 3: Characterization of CWC22 domains and binding sites that mediate interaction with AdML pre-mRNA.

(A) *In vitro* splicing and mRNP-immunoprecipitation of AdML-PT60 pre-mRNA in the presence of FLAG-tagged CWC22 deletion mutants was performed as described in Supplementary Figure 1.

(B) Quantification of AdML-PT60 intron-lariat mRNA precipitated by the indicated FLAG-tagged CWC22 proteins compared to the reaction input. Error bars = SD, N = 4. FLAG-CWC22 and FLAG-CWC22 (110-665) precipitated significantly more mRNA than the negative control ($p = 0.0063$ and $p = 0.048$).

(C) *In vitro* splicing and mRNP immunoprecipitation of AdML-PT60 pre-mRNA in the presence of the indicated FLAG-tagged CWC22 mutants was performed as described in Figure 1.

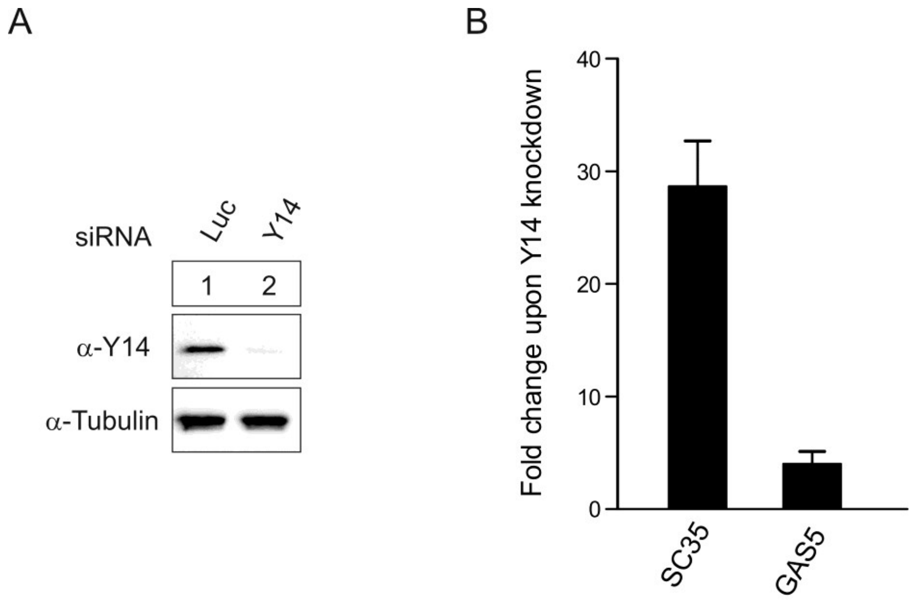

Supplementary Figure 4: Knockdown of the EJC-protein Y14 causes an upregulation of endogenous NMD targets.

(A) Expression of Y14 in extracts from HEK293 cells that were transfected with siRNA targeting Y14 or luciferase, as detected by immunostaining with a specific antibody. Tubulin served as a loading control.

(B) qPCR analysis determining the fold change in SC35 (1.7kb splice product) and GAS5 mRNA levels upon Y14-depletion in HEK293 cells. Expression levels were normalized to the housekeeping gene TATA-box binding protein (TBP). Error bars = SD, n=2.

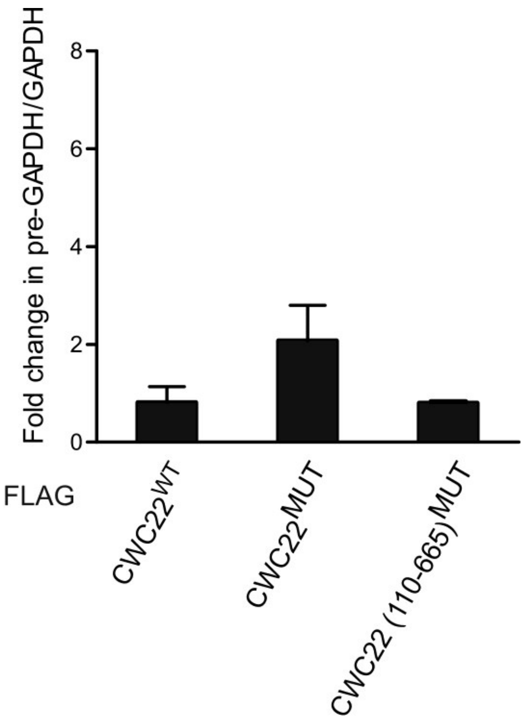

Supplementary Figure 5: Overexpression of CWC22^{mut} causes only a modest change in pre-mRNA splicing.

qPCR analysis determining the fold change in the GAPDH pre-mRNA/mRNA ratio upon overexpression of the indicated CWC22 proteins in HEK293 Flp-In cells. Error bars = SD, n = 4.

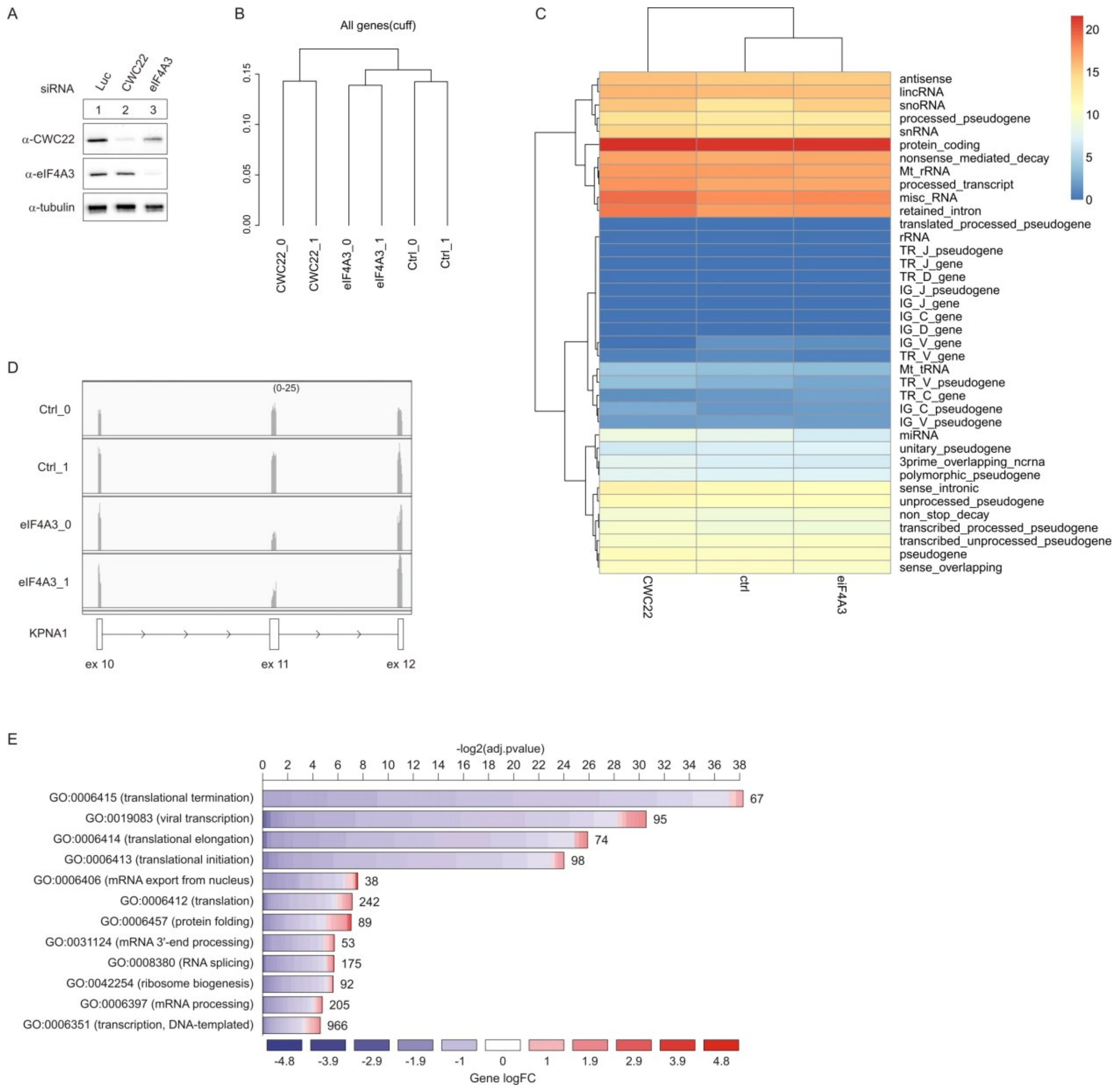

Supplementary Figure 6: RNA-seq analysis of cells depleted of CWC22 or eIF4A3.

(A) Immunoblot showing the protein expression in cells analyzed by RNA-seq. HeLa cells were transfected with the indicated siRNAs and harvested in Isol-RNA lysis reagent 48 hours after transfection. Proteins were extracted from the phenole phase and expression levels were determined by immunostaining with specific antibodies. Tubulin served as a loading control

(B) Hierarchical clustering of gene expression values over all replicate samples. The underlying distance matrix to generate the dendrogram was computed from the Jensen-Shannon distance over all pairs of gene expression probability vectors.

(C) Reads mapping KPNA1 exon 10, 11 and 12 visualized in the integrated genome viewer (IGV). Scheme at the bottom of the panel depicts the exon-intron structure of the KNPA1 gene. The maximum of the Y-axis (number of mapped reads) was set to 25.

(D) Enrichment of gene onthology (GO) terms in the group of regulated genes upon CWC22 depletion. The width of the bars represents the significance (–log2(adjusted p-value)) of the respective GO term enrichment. Colors depict the log fold change (log FC) of individual genes within the GO category and numbers behind the bars correspond to the number of genes within the GO category.

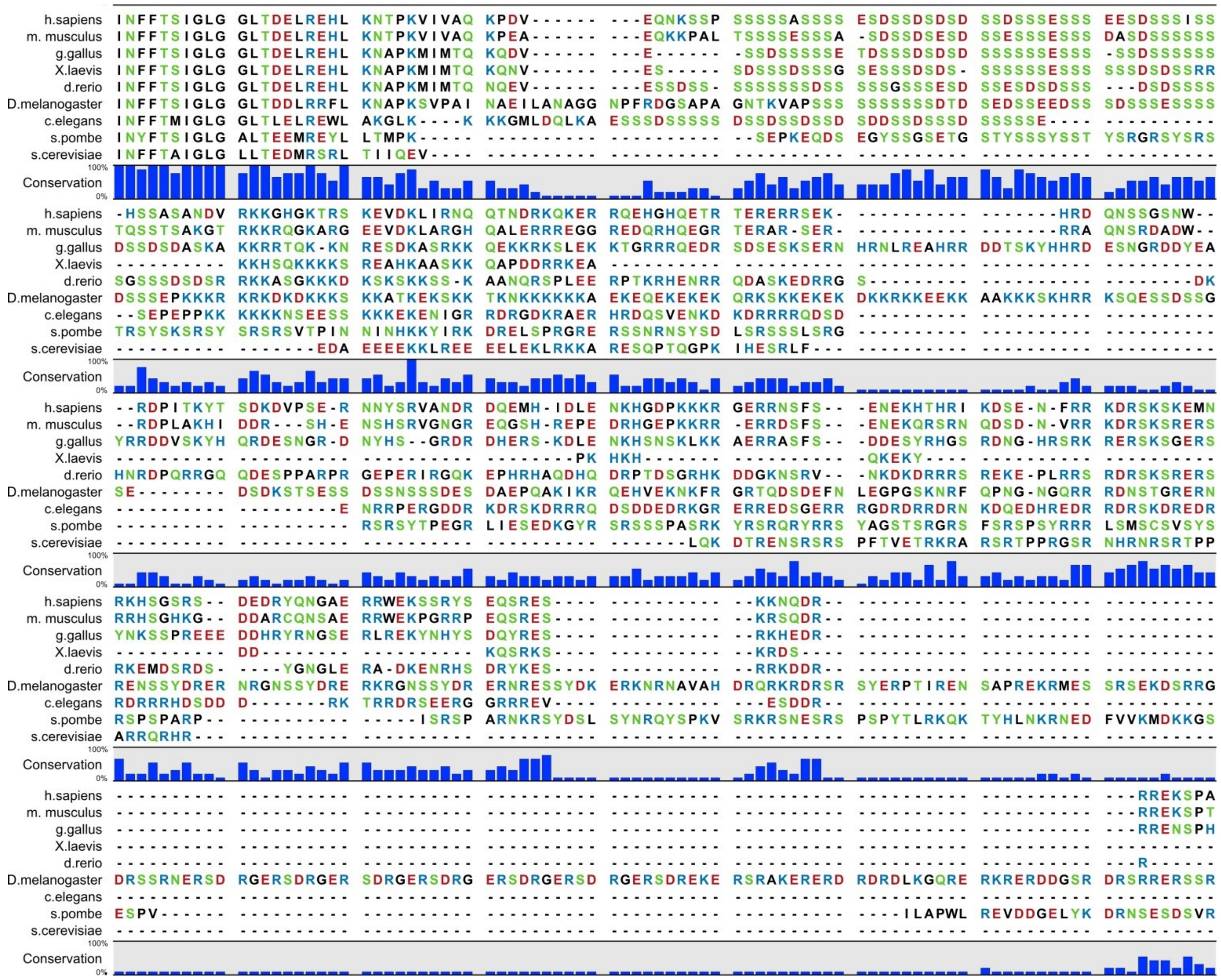

Supplementary Figure 7: Alignment of the C-terminal domain of CWC22 from different species. The alignment was generated using CLC workbench (qiagen).
